# Maternal diet-induced obesity programmes cardiac dysfunction in male mice independently of post-weaning diet

**DOI:** 10.1093/cvr/cvy082

**Published:** 2018-04-04

**Authors:** Elena Loche, Heather L Blackmore, Asha A Carpenter, Jessica H Beeson, Adele Pinnock, Thomas J Ashmore, Catherine E Aiken, Juliana de Almeida-Faria, Josca M Schoonejans, Dino A Giussani, Denise S Fernandez-Twinn, Susan E Ozanne

**Affiliations:** 1 University of Cambridge Metabolic Research Laboratories and MRC Metabolic Diseases Unit, Wellcome Trust-MRC Institute of Metabolic Science, Level 4, Box 289, Addenbrookes' Treatment Centre, Addenbrookes' Hospital, Hills Road, Cambridge, CB2 OQQ, UK; 2 Department of Obstetrics and Gynaecology, The Rosie Hospital and NIHR Cambridge Comprehensive Biomedical Research Centre, University of Cambridge, Box 223, Cambridge, CB2 0SW, UK; 3 University of Campinas, Faculty of Medical Sciences, Obesity and Comorbidities Research Center, Campinas, 13083-864, Brazil; 4 Department of Physiology, Development & Neuroscience, University of Cambridge, Downing Street, Cambridge, CB2 3EG, UK

**Keywords:** Maternal diet-induced obesity, Pregnancy, Cardiovascular disease, Developmental programming

## Abstract

**Aims:**

Obesity during pregnancy increases risk of cardiovascular disease (CVD) in the offspring and individuals exposed to over-nutrition during fetal life are likely to be exposed to a calorie-rich environment postnatally. Here, we established the consequences of combined exposure to a maternal and post-weaning obesogenic diet on offspring cardiac structure and function using an established mouse model of maternal diet-induced obesity.

**Methods and results:**

The impact of the maternal and postnatal environment on the offspring metabolic profile, arterial blood pressure, cardiac structure, and function was assessed in 8-week-old C57BL/6 male mice. Measurement of cardiomyocyte cell area, the transcriptional re-activation of cardiac fetal genes as well as genes involved in the regulation of contractile function and matrix remodelling in the adult heart were determined as potential mediators of effects on cardiac function. In the adult offspring: a post-weaning obesogenic diet coupled with exposure to maternal obesity increased serum insulin (*P *<* *0.0001) and leptin levels (*P *<* *0.0001); maternal obesity (*P *=* *0.001) and a post-weaning obesogenic diet (*P *=* *0.002) increased absolute heart weight; maternal obesity (*P *=* *0.01) and offspring obesity (*P *=* *0.01) caused cardiac dysfunction but effects were not additive; cardiac dysfunction resulting from maternal obesity was associated with re-expression of cardiac fetal genes (*Myh7: Myh6* ratio; *P *=* *0.0004), however, these genes were not affected by offspring diet; maternal obesity (*P *=* *0.02); and offspring obesity (*P *=* *0.05) caused hypertension and effects were additive.

**Conclusions:**

Maternal diet-induced obesity and offspring obesity independently promote cardiac dysfunction and hypertension in adult male progeny. Exposure to maternal obesity alone programmed cardiac dysfunction, associated with hallmarks of pathological left ventricular hypertrophy, including increased cardiomyocyte area, upregulation of fetal genes, and remodelling of cardiac structure. These data highlight that the perinatal period is just as important as adult-onset obesity in predicting CVD risk. Therefore, early developmental periods are key intervention windows to reduce the prevalence of CVD.

## 1. Introduction

Obesity has reached epidemic proportions worldwide, and for the first time more people in the world are obese than underweight.^1^ Obesity is a risk factor for cardiovascular disease (CVD), which remains the most common cause of death in Europe.^2^ Recent statistics have highlighted that the prevalence of obesity among women of reproductive age is dramatically increasing, with the UK demonstrating the highest prevalence in Europe (prevalence of 25.2%).^3^ This is a major concern, as maternal obesity is detrimental not just for the mother, as it associates with higher risk of developing gestational diabetes, pre-eclampsia, and miscarriage, but also for her baby in the short (e.g. congenital anomalies, stillbirth) as well as in the long-term.^4^ This has been termed ‘developmental programming’.^5^

The concept that the environment experienced by an individual during early life can shape its long-term health through developmentally programmed effects forms the basis of the Developmental Origins of Health and Disease (DOHaD).^5^ A wealth of studies in humans and animal models has shown that environmental insults during critical periods of development (e.g. suboptimal maternal nutrition, maternal stress, placental dysfunction, chronic fetal hypoxia) drive structural and metabolic alterations in the conceptus at the multi-organ level, leading to an increased risk of obesity, type-2 diabetes, and CVD.^6^^,^^7^ The fetal environment associated with maternal obesity has recently been shown to be one of those associated with increased risk of such diseases.

Maternal obesity during pregnancy increases the long-term risk of cardiometabolic disease in the offspring.^8^ The offspring cardiovascular system is particularly vulnerable to suboptimal exposures, such as maternal over-nutrition during gestation and lactation. In rodents, sheep and non-human primates, offspring of over-nourished dams are more prone than their control counterpart to cardiovascular pathology such as endothelial dysfunction,^9^ cardiac hypertrophy,^10^ hypertension,^11^^,^^12^ fibrosis,^13^ and impaired left ventricular function.^14^ Similarly, offspring born to dams fed either a high-fat or obesogenic (high-fat high-sugar) diet prior to and during gestation develops metabolic perturbations, including hyperinsulinaemia in the presence^15^ or absence^16^^,^^17^ of increased fasting glucose levels. Human epidemiological studies support the animal evidence. Maternal body mass index positively correlates with premature death from cardiovascular events in the offspring.^18^^,^^19^ In addition, bariatric surgery in obese women improves insulin sensitivity, reduces adiposity and blood pressure in individuals born after maternal surgery compared to their siblings born pre-surgery.^20^ These studies strongly support a pivotal role of the maternal environment in the programming of cardiometabolic diseases in the offspring. Indeed, human fetuses of obese mothers develop signs of cardiac dysfunction and insulin resistance *in utero*.^21^^,^^22^

Risk of CVD depends not only on fetal and early life exposure to a suboptimal maternal diet but also on the postnatal environment. Children born to obese women are more likely to be exposed to the same obesogenic environment growing up,^23^ which implies that CVD risk may be further modified by the quality of postnatal life. In rodents, a post-weaning obesogenic diet has been reported to exacerbate the detrimental effects of maternal diet-induced obesity on offspring body weight gain,^24^^,^^25^ lipid metabolism,^26^ insulin sensitivity,^16^^,^^27^ and endothelial dysfunction.^28^ However, whether an additional exposure to a calorie-rich postnatal environment worsens the programmed cardiac effects on the adult offspring of a maternal obesogenic diet is relatively unexplored in any species.

Using a well-established mouse model of maternal diet-induced obesity, we have previously shown that mice born to over-nourished mothers but fed a chow diet post-weaning develop left ventricular hypertrophy (LVH) and systolic and diastolic dysfunction in adulthood.^14^^,^^29^ LVH is defined by an increase in left ventricular cardiomyocyte size rather than number.^30^ Although initially adaptive, LVH may become pathological if the trigger signal persists, overwhelming compensation and ultimately leading to matrix remodelling, impaired contractility and heart failure.^31^ A hallmark of pathological hypertrophy is the transcriptional re-activation of cardiac fetal genes in the adult heart,^32^ among which changes in the expression of genes encoding for the natriuretic peptides A (*Nppa)* and B *(Nppb)*, alpha-skeletal actin (*Acta1*), and a shift from the predominant adult-fast myosin α- (*Myh6*) to the fetal form β-myosin heavy chain (*Myh7*) occur, which lowers contractile capacity.^32^ Similarly, dysregulation of genes in the calcium signalling pathway (sarco/endoplasmic reticulum Ca^2+^-ATPase [*Serca2a*], cardiac muscle troponin T [*Tnnt2*]),^33^ and matrix remodelling (collagen Type I Alpha 1 [*Col1a1*], collagen Type III Alpha 1 [*Col3a1*])^34^ have been reported in cardiac hypertrophic disease and these changes contribute to alterations in cardiac contractility.

The effects of weaning onto an obesogenic diet on offspring metabolism, cardiac structure, *in vivo* cardiac function, and blood pressure following maternal diet-induced obesity are not known. Therefore, in this study, we determined the effects of a combined exposure to a maternal and a post-weaning obesogenic environment, and their interaction, on whole-body metabolism, arterial blood pressure, cardiac structure, and function in young adult male mice. Body fat and lean mass as well as plasma leptin, insulin, glucose, total cholesterol, triglycerides, and free fatty acids were determined in the adult offspring. Molecular mechanisms that could mediate changes in cardiac structure and function in the adult murine heart were addressed, including the transcriptional re-activation of cardiac fetal genes as well as genes involved in the regulation of contractile function and matrix remodelling.

## 2. Methods

### 2.1 Animal work

This research has been regulated under the Animals (Scientific Procedures) Act 1986 Amendment Regulations 2012, which transposed Directive 2010/63/EU into UK law, following ethical review by the University of Cambridge Animal Welfare and Ethical Review Body (AWERB). A detailed diagram of the animal model is reported in Supplementary material online, *Figure S1*. Female C57BL/6 mice, ∼4 weeks of age were fed *ad libitum* either a standard control chow diet (RM1, 7% simple sugars, 3% fat, 15% protein [w/w], 3.5 kcal/g, Special Dietary Services, Dietex International Ltd., Witham, UK) or a highly palatable energy-rich obesogenic diet (10% simple sugars, 20% animal fat, 23% protein [w/w], 4.5 kcal/g, Special Dietary Services) and sweetened condensed milk (55% simple sugar, 8% fat, 8% protein [w/w], 3.2 kcal/g, Nestle, UK) fortified with mineral and vitamin mix (AIN93G, Special Dietary Services). A detailed composition of the diets has been described previously.^12^ Dams were maintained on their respective diets pre-gestation, during gestation and lactation, and were mated with chow-fed males. Dams were allowed to litter and the first litter culled after weaning. This first pregnancy ensured the mice were proven breeders. After a week, mice were re-mated for a second pregnancy and day 1 of pregnancy was defined by the appearance of a post-copulatory plug. Forty-eight hours after delivery of the second litter, the number of pups in each litter was normalized to six to avoid confounding effects of litter size during lactation. At day 21, male pups were randomly divided by a technician (blinded to the scientific objectives) into four experimental groups: male offspring of control mums weaned either onto a chow diet (CC) or an obesogenic diet (CO), and male offspring of obese mothers weaned onto either a chow diet (OC) or an obesogenic diet (OO) (see Supplementary material online, *Figure S1*). Offspring were killed at 8 weeks of age by rising CO_2_ concentration following a 4-h fast (8.30–12.30 am). Only male offspring were studied to avoid the confounding influences of sexual dimorphism, and only one male mouse per litter was used in each analysis. To prevent confounding effects, tissues for molecular analysis were harvested from mice that did not undergo any other experimental procedure. Blood was collected by cardiac puncture, allowed to clot, and serum obtained by two centrifugation steps (both 3000 *g* for 3 min) to ensure complete removal of red blood cells; sera were frozen at −80°C for later analyses. Tissues were collected, weighed, and frozen at −80°C. Mice were housed in pairs or trios in individually ventilated cages and maintained in a 12-h light/dark cycle at a housing temperature of ∼23°C. Drinking water and diets were provided *ad libitum*. Food intake was measured by weekly weighing of food pellets remaining in the hopper from the previous measurement, with fresh diet additions accounted for.

### 2.2 Serological analyses

A range of metabolites and hormones were measured in serum. Levels of insulin and leptin were measured using the Ultra-Sensitive Mouse Insulin ELISA Kit (Crystal Chem, USA) and Mouse Leptin ELISA kit (Crystal Chem) according to the manufacturer’s instructions. Total cholesterol, triglycerides, free fatty acids, adiponectin, resistin, and visfatin were measured by the MRC MDU Mouse Biochemistry Laboratory (Addenbrookes Hospital, Cambridge, UK). HOMA-IR was calculated from the fasting blood glucose (mmol/L) × fasting plasma insulin (µU/ml) divided by 22.5.

### 2.3 Adipose tissue isolation

Brown adipose tissue (BAT) was located above the scapulae; the butterfly shaped BAT depot was carefully excised and any superficial white adipose tissue removed. Subcutaneous adipose tissue located in the dorsal lumbar part, between the skin and the abdominal wall, was isolated.

### 2.4 RNA extraction

Total RNA was extracted from cardiac and adipose tissues using a miRNeasy mini kit (Qiagen Ltd., UK). Powdered tissue (25 mg) was homogenized in 700 μL of Qiazol using TissueRuptor (Qiagen), and the protocol was followed according to the manufacturer’s instructions. RNA was eluted in 30 μL RNase-free H_2_O and quantified by spectrophotometric analysis (NanoDrop 2000, ThermoFisher, UK). RNA quality was assessed by determination of the integrity of the 28S and 18S ribosomal RNA bands following electrophoresis on a 1% agarose gel. Samples were stored at −80°C until use.

### 2.5 Complementary DNA synthesis and quantitative real-time–PCR

Complementary DNA (cDNA) was generated from 400 ng total RNA per sample using the High Capacity cDNA Reverse Transcription Kit (Applied Biosystems, ThermoFisher, UK) with random primers. cDNA samples were diluted 1:20 and real-time quantitative PCR was performed using SYBR Green PCR Master Mix (Applied Biosystems) and synthetic oligonucleotides as primers (see Supplementary material online, *Table S1*). Primers were designed to assay all annotated splice-variants of a gene where possible, and were checked for specificity using NCBI nucleotide BLAST. A six-point standard curve of two-fold dilution was prepared from pooled cDNA to assess amplification efficiency of primer pairs. All primers amplified with an estimated efficiency between 90% and 110%, and there was no evidence of inhibitors present in the reaction. Quantification was performed using the comparative Ct method.^35^ Target gene expression was normalized to the geometric mean of *Gapdh* and *Sdha* (cardiac tissue) and to *36b4* (subcutaneous and brown adipose tissue). The expression levels of the genes of reference did not differ between groups. Statistics were performed on the relative fold change (2^−ΔΔCT^).

### 2.6 Cardiac stereology

Hearts were fixed in 10% neutral buffered formalin, processed, and embedded in paraffin. Serial sections of 10 µM were cut using a Leica RM2235 (Leica Biosystems, UK) rotary microtome in the long axis. Selected slides were stained with haematoxylin and eosin. Sections were visualized using a BX-50 microscope (Olympus, UK) fitted with a motorized specimen stage and microcator. Analyses were performed blinded using the Computer Assisted Stereology Toolbox version 2.0 (Olympus) and the Cavalieri principle (point-counting method) as previously described.^36^

### 2.7 Cardiomyocyte cell size

Wheat germ agglutinin was used to stain cardiomyocyte cell borders using a previously described method [Texas Red-X conjugate, (Molecular Probes, Life Technologies)].^29^ Mid-cardiac sections (*n* = 3 slides per animal, *n* = 6 animals per dietary group) of formalin-fixed paraffin embedded hearts were de-waxed by immersion in 100% xylene (3 times for 2 min), followed by 100% ethanol (2 times for 2 min), and a final rinse in H_2_O. Slides were incubated with 10 μg/mL of conjugated agglutinin (diluted in PBS) while gently rocking in the dark for 2 h at room temperature. Slides were then washed with PBS and air-dried before addition of Vectashield mounting medium with 4′,6-diamidino-2-phenylindole (Vector Laboratories). Images of the left ventricular wall were taken with a 40× objective on the confocal laser-scanning platform TCS Leica SP8 (Leica Microsystems, Germany), and analysed double-blinded using a semi-automated pipeline on the open source program CellProfile. Cell area was calculated as described previously^14^ and data were analysed using a hierarchical linear model with random effects for both individual animal and an interactive effect between individual and section of origin. The model included maternal diet and offspring diet as fixed effects. This structure accounted for the fact that multiple cell area measurements were obtained from multiple sections (*n* = 3) of the heart of each individual animal within the dietary groups, and these data cannot be treated as fully independent.

### 2.8 Assessment of *in vivo* cardiac function

Trans-thoracic echocardiography to assess left ventricular function was performed using the VisualSonics Vevo 770 high‐resolution imaging system equipped with a 30‐MHz RMV‐707B probe. Mice were anaesthetized with isoflurane (1.5% v/v after initial induction at 2% v/v) by inhalation, and imaged in the supine position while placed on a heated platform. Heart rate was monitored via electrocardiography electrodes built into the platform and temperature via a rectal probe. The parasternal long axis view of the left ventricle was achieved in B-mode at the level of the papillary muscles. M-mode images were subsequently obtained by placing a vertical cursor in the middle of the ventricle on the moving B-mode image. Wall dimensions in both systole and diastole were calculated using the Vevo 770 software analysis package, these included: left ventricular posterior wall (LVPW) and left ventricular internal diameter (LVID). Functional parameters including ejection fraction (EF), stroke volume (SV), cardiac output (CO), heart rate (HR) were calculated using the M-mode LV tracing tool on the Vevo 770 analysis software, whereby a trace was made of both the upper and lower wall across a minimum of four cardiac cycles. Volumetric measurements (at the end of diastole and systole) were also calculated using this tool. Cardiac output was automatically derived using the calculation CO = SV x HR. Volumetric measurements were calculated using the Teichholz method. 

### 2.9 Assessment of cardiac fibrosis

Paraffin embedded ventricular mid-cardiac sections (10 µM) were stained with Picrosirius Red (Pioneer Research Chemicals Ltd, Essex, UK). Areas positive for fibrosis stained red. Per section, eight images across the ventricles were randomly selected using a grid and random number generator system. Total fibrosis, including perivascular fibrosis, was calculated as a percentage of tissue area using the threshold system of Fiji-Image J. The same threshold was applied across all images and groups. 

### 2.10 Lipid peroxidation assay

Lipid Peroxidation Assay Kit (Abcam, ab118970) was used to quantify malondialdehyde (MDA) concentration in 10 mg of cardiac tissue following manufacturer’s instructions. Data were normalized to tissue weight.

### 2.11 Blood pressure measurement

Non-invasive blood pressure was measured by restraint tail cuff photoplethysmography using a BP-2000 Series II System (Visitech Systems, USA) in the same animals used for echocardiography prior to the assessment of cardiac function. Blood pressure was measured between 4 and 5 pm. Mice were trained over 2 days to familiarize them with the apparatus and provide more reproducible results. On the third measurement day blood pressure measurements had reached a stable level and actual experimental recording was performed.

### 2.12 Body composition analysis

Total body fat and lean mass of control and obese dams was determined at the time of mating using Time-Domain Nuclear Magnetic Resonance (TD-NMR), which is a non-invasive method of imaging of live conscious mice at weekly intervals. Similar measurements on the 8-week-old offspring were made on the day of tissue collection. Data are reported as absolute fat or lean tissue mass in grams. 

### 2.13 Statistical analysis

Data were analysed using Prism 6 (GraphPad). Offspring growth trajectory was plotted as an average of individual measurements (*n* = 8) at each time point. Only one male mouse from each litter was used in each analysis as the experimental unit in programming studies is the dam (i.e. eight pups = eight dams represented). Statistical comparisons were made using an unpaired two-tailed Student’s *t*-test, or a two-way analysis of variance (ANOVA) to estimate the effect of two variables (i.e. maternal diet and offspring diet), followed by Dunnet’s multiple comparison test. The hierarchical linear model was computed using the R statistical software package version 2.14.1 (R Foundation for Statistical Computing, Vienna, Austria). Data are means ± SEM unless stated otherwise. For all statistical comparisons, the level of significance was set at *P *<* *0.05.

## 3. Results

### 3.1 Effects of maternal obesity and post-weaning obesogenic diet on body weight and heart weight

Dams fed the obesogenic diet were significantly heavier than dams fed the control diet at mating (33.5 ± 1.3 g vs. 26.4 ± 0.9 g; *P *<* *0.0001) (see Supplementary material online, *Figure S2A*), had increased fat mass (11.08 ± 0.3 g vs. 3.5 ± 0.3 g; *P *<* *0.0001) (see Supplementary material online, *Figure S2B*) and were hyperphagic throughout pregnancy (620.9 ± 98 kcal consumed vs. 359.2 ± 25.3 kcal consumed; *P *<* *0.05) (see Supplementary material online, *Figure S2C*). Pups from obese mums showed no difference in body weight at day 2. However, obese pups were significantly heavier than control pups on day 14 (8.8 ± 0.7 g vs. 6.8 ± 0.1 g*; P *=* *0.02) and remained heavier at weaning (11.5 ± 0.9 g vs. 8.5 ± 0.3 g*; P *=* *0.006) (see Supplementary material online, *Figure S2D*).

**Figure 1:**
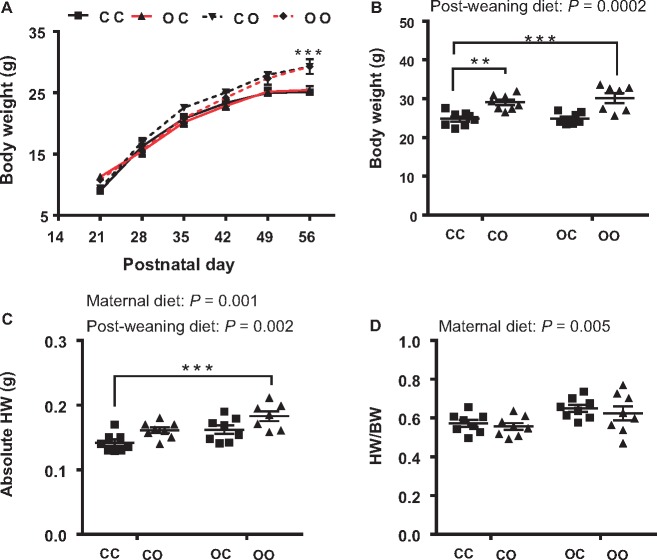
Exposure to maternal over-nutrition is sufficient to increase heart weight. (*A*) Offspring growth curve from weaning to 8 weeks (in red are groups exposed to maternal obesity); (*B*) body weight at 8 weeks of age; (*C*) absolute heart weight; (*D*) heart weight normalized to body weight. *N* = 7–8 male mice from different dams were used for each group. CC, mum fed chow, offspring fed chow; OC, mum fed obesogenic diet, offspring fed chow; CO, mum fed chow, offspring fed obesogenic diet; OO, mum fed obesogenic diet, offspring fed obesogenic diet. Two-way ANOVA followed by Dunnet’s *post hoc* test for multiple comparisons vs. CC was performed. *P < 0.05, **P < 0.01, ***P < 0.001.

At 8 weeks of age, male mice fed the post-weaning obesogenic diet were significantly heavier than those weaned onto chow (two-way ANOVA, effect of offspring diet *P *=* *0.0002) (*Figure 1A* and *B*). However, there was no effect of maternal diet on offspring body weight at this age. Consistent with our previous findings,^14^^,^^29^ offspring exposed to over-nutrition during gestation and lactation showed an increase in heart weight (two-way ANOVA, effect of maternal diet *P *=* *0.001) (*Figure 1C*). A post-weaning obesogenic diet also caused an increase in absolute heart weight (effect of offspring diet *P *=* *0.002). The OO group had the highest absolute heart weight (23% increase vs. CC) (*Figure 1C*) suggesting that the effects of maternal diet and offspring diet were additive. A significant effect of maternal diet was also observed on heart weight relative to body weight (two-way ANOVA, *P *=* *0.005) (*Figure 1D*) suggesting a profound influence of the maternal diet on offspring cardiac size at 8 weeks of age. There was no effect of offspring diet on relative heart weight suggesting that the post-weaning obesogenic diet increased heart weight in proportion to body weight.

### 3.2 Effects of maternal obesity and post-weaning obesogenic diet on 8-week-old offspring

Offspring born to over-nourished mothers showed increased total adiposity (two-way ANOVA, effect of maternal diet *P *=* *0.0195), and a post-weaning obesogenic diet also increased fat mass (two-way ANOVA, effect of offspring diet *P *=* *0.0005). These effects were additive as reflected by the observation that the OO group had the greatest fat mass (two-way ANOVA, *post hoc P *<* *0.001) (*Table 1*). Visceral fat distribution was strongly affected by post-weaning diet but there was no significant effect of maternal diet, either when expressed as absolute weight or relative to body weight (see Supplementary material online, *Table S2*). Offspring of obese mothers had significantly heavier brown adipose tissue (BAT) weights compared to control groups (see Supplementary material online, *Table S2*). This observation is consistent with a previous study showing that offspring born to high-fat diet-fed dams had greater BAT mass at weaning.^37^ BAT weight was also affected by a post-weaning obesogenic diet (*P *<* *0.0001) as previously reported.^38^ The expression of subcutaneous or brown fat uncoupling protein 1 (*Ucp1*), peroxisome proliferator-activated receptor gamma coactivator 1-alpha (*Pgc1a*), peroxisome proliferator-activated receptor alpha (*Ppara*), and peroxisome proliferator-activated receptor gamma (*Pparg*) were not different between any of the groups (see Supplementary material online, *Figure S3*). Leptin (*P *<* *0.0001) and resistin (*P = *0.0254) levels were increased by offspring diet but were not influenced by maternal diet. No effects on adiponectin levels were observed in any of the groups. While maternal obesity marginally increased insulin concentration in the offspring (two-way ANOVA, *P *=* *0.076), a post-weaning high-fat diet resulted in highly elevated serum insulin levels (two-way ANOVA, effect of offspring diet *P *<* *0.0001). Blood glucose levels were not affected by maternal diet or offspring diet (*Table 1*). Therefore, the increase in insulin in the absence of any change in glucose concentrations suggests that both maternal obesity and an offspring obesogenic diet independently led to insulin resistance. A post-weaning obesogenic diet caused a significant increase in total cholesterol (two-way ANOVA, *P *=* *0.0004), whereas no significant difference was observed in triglyceride levels. There was no effect of maternal diet on cholesterol or triglyceride levels. There was no significant interaction between maternal and offspring diet on any of these metabolic parameters, suggesting all these effects were independent and therefore additive.

**Table 1:** A maternal and post-weaning obesogenic diet have additive effects on offspring fat mass, insulin, and leptin levels

	CC	CO	OC	OO	MD	PD	MDxPD
					*P values*		
Fat mass (g)	2.5±0.3	3.7±0.7	2.9±0.2	5.6±0.7***	0.0195	0.0005	0.1195
Lean mass (g)	19.4±0.6	21.1±0.6	18.9±0.4	21.7±0.5*	0.9149	0.0012	0.4210
Insulin (pmol/L)	127±11	422±109*	166±14	663±97***	0.0763	<0.0001	0.1940
Leptin (ng/mL)	1.2±0.08	9.9±2.7*	2.2±0.2	13.6±2.8***	0.2707	<0.0001	0.5242
Adiponectin (µg/mL)	24.9±0.5	25.3±1.5	27.9±1.5	24.2±1.6	0.5321	0.2620	0.1704
Resistin (ng/mL)	21.6±0.6	26.9±2.4	24.7±1.3	28.6±2.5	0.2313	0.0254	0.7049
Glucose (mmol/L)^a^	11.9±0.5	11.9±0.8	11.1±0.7	12.3±1.2	0.7753	0.5038	0.5038
HOMA-IR	11.4±1.3	40.9±11.9	15.2±1.8	63.7±12.9***	0.1459	0.0001	0.2933
HDL (mmol/L)	1.88±0.04	2.52±0.14*	1.85±0.13	2.27±0.22	0.3717	0.0018	0.4995
LDL (mmol/L)	0.78±0.04	1.24±0.12**	0.70±0.11	1.04±0.07	0.1596	0.0003	0.5822
Cholesterol (mmol/L)	3.21±0.09	4.25±0.2**	3.29±0.09	4.05±0.33*	0.7799	0.0004	0.5481
FFA (µmol/L)	1302±40	1117±68	1257±102	1168±83	0.9629	0.0780	0.5247
TG (mmol/L)	1.3±0.09	1.1±0.06	1.3±0.09	1.3±0.11	0.2369	0.3633	0.1132

Quantification of circulating metabolites on ^a^tail blood and sera collected after a 4-h fast in 8-week-old male offspring. *N*= 6–8 animals from independent litters for each dietary group. Two-way ANOVA followed by Dunnett’s *post hoc* test for multiple comparisons vs. CC was performed to calculate the effect of maternal diet (MD), postnatal diet (PD), and the interaction between the two (MDxPD).

CC, mum fed chow, offspring fed chow; OC, mum fed obesogenic diet, offspring fed chow; CO, mum fed chow, offspring fed obesogenic diet; OO, mum fed obesogenic diet, offspring fed obesogenic diet; HDL, high-density lipoprotein; LDL, low-density lipoprotein; FFA, free fatty acids; TG, triglycerides.

Dunnett’s multiple comparisons test was then performed to evaluate significant differences to the CC group: *P < 0.05, **P < 0.01, ***P < 0.001.

### 3.3 Maternal over-nutrition programmes pathological cardiac hypertrophy in the offspring

**Figure 2:**
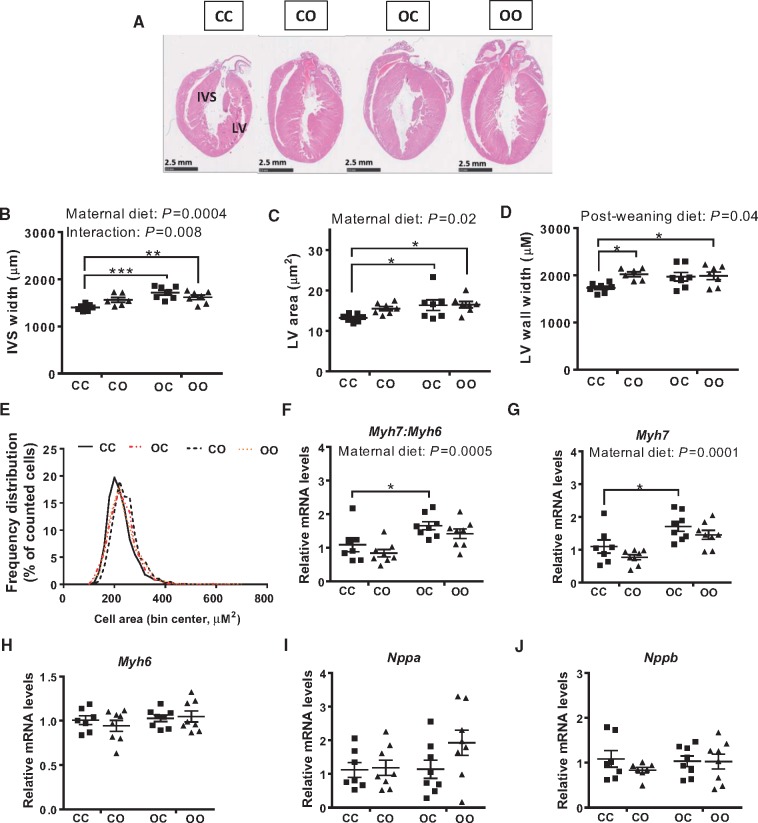
Exposure to maternal over-nutrition programmes pathological cardiac hypertrophy. (*A*) Representative H&E staining of mid-cardiac sections; (*B*) interventricular septum (IVS) width; (*C*) area of the left ventricle; (*D*) left ventricular (LV) wall width. *N*= 6–7 animals from independent litters for each dietary group. (*E*) Frequency distribution of cardiomyocytes cell area in the left ventricle of CC, OC, CO, and OO 8-week-old animals (in red are groups exposed to maternal obesity; *n* = 6/dietary group); (*F–J*) mRNA levels of cardiac hypertrophic markers *Myh7: Myh6*, *Myh7*, *Myh6*, *Nppa*, and *Nppb* (*n* = 7–8). CC, mum fed chow, offspring fed chow; OC, mum fed obesogenic diet, offspring fed chow; CO, mum fed chow, offspring fed obesogenic diet; OO, mum fed obesogenic diet, offspring fed obesogenic diet. Two-way ANOVA followed by Dunnett’s *post hoc* test for multiple comparisons vs. CC was performed. The overall effect of maternal diet and post-weaning diet is reported where significant in each panel. Significant vs. CC: *P < 0.05, **P < 0.01, ***P < 0.001.

We measured cardiac dimensions in the four experimental groups by stereology. Mid-transverse sections showed that the OC, CO, and OO groups all had larger hearts compared to the CC group, with OO mice having the largest hearts (*Figure 2A*). Stereological analysis of whole hearts revealed maternal diet significantly increased interventricular septum width (two-way ANOVA, effect of maternal diet *P *=* *0.0004) (*Figure 2B*) and left ventricular area (two-way ANOVA, effect of maternal diet *P *=* *0.02) (*Figure 2C*) but these measurements were not affected by offspring diet. Left ventricular free wall width was increased by offspring diet (two-way ANOVA, *P *=* *0.04) (*Figure 2D*), but not significantly by maternal diet. Cardiomyocyte cell area was increased in CO (241.5 ± 1.4 µm^2^), OC (230.3 ±1.4 µm^2^), and OO (232.5 ±1.5 µm^2^) animals compared to CC (222.9 ±1.4 µm^2^) (*Figure 2E*), and statistical analysis using a hierarchical linear model revealed a significant effect of maternal diet (*P *<* *0.001). Overall, maternal diet-induced obesity led to an increased ratio of *Myh7: Myh6* (two-way ANOVA, *P *=* *0.0005) (*Figure 2F*). The expression of *Myh7: Myh6* was not affected by an offspring obesogenic diet. The increase in *Myh7: Myh6* was driven by up-regulation of *Myh7* (*Figure 2G*). No significant difference between any of the groups was observed in the expression of *Myh6* (*Figure 2H*), *Nppa* (*Figure 2I*), or *Nppb* (*Figure 2J*).

### 3.4 Maternal over-nutrition programmes cardiac dysfunction

**Figure 3:**
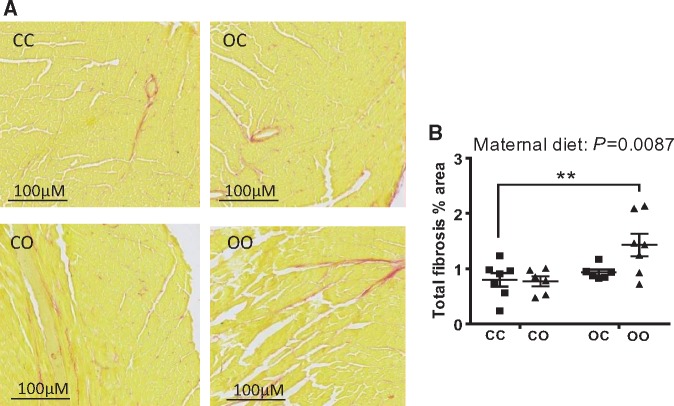
Male offspring exposed to maternal obesity develop signs of cardiac fibrosis. (*A*) Representative images of ventricular mid-cardiac sections (10 µm) stained with Picrosirius Red. Areas positive for fibrosis were stained red; (*B*) quantification of total fibrosis expressed as a percentage of tissue area. CC, mum fed chow, offspring fed chow; OC, mum fed obesogenic diet, offspring fed chow; CO, mum fed chow, offspring fed obesogenic diet; OO, mum fed obesogenic diet, offspring fed obesogenic diet. Data are presented as means ± SEM (*n* = 6–8 from independent litters for each dietary group) and were analysed by two-way ANOVA. Dunnett’s multiple comparisons test was performed to evaluate significant differences to the CC group: **P < 0.01.

**Figure 4:**
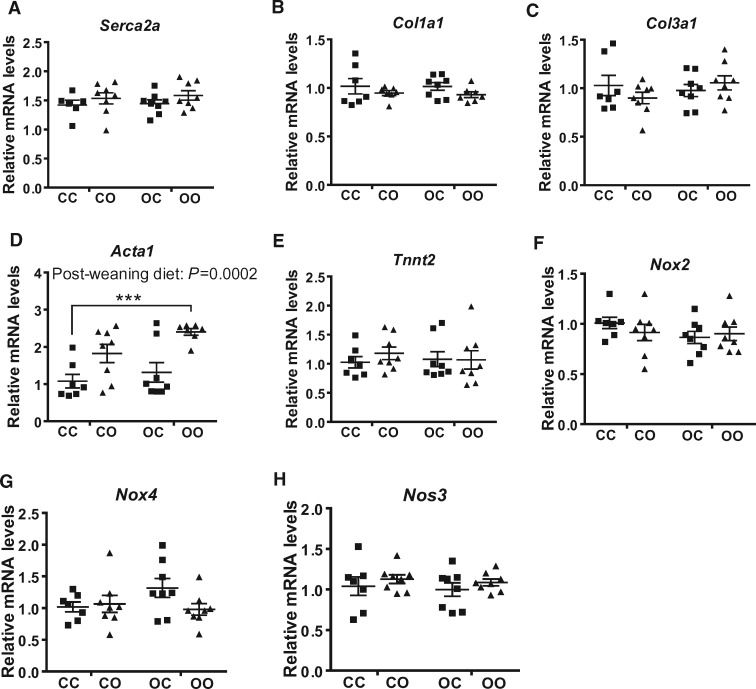
Maternal obesity only partially affects the expression of contractile and matrix remodelling genes in offspring hearts. Gene expression of collagen encoding for *Serca2a* (*A*), *Col1a1* (*B*), *Col3a1* (*C*), *Acta1* (*D*), *Tnnt2* (*E*), *Nox2* (*F*), *Nox4* (*G*), and *Nos3* (*H*) in the cardiac tissue of the four experimental groups, expressed relative to CC group. CC, mum fed chow, offspring fed chow; OC, mum fed obesogenic diet, offspring fed chow; CO, mum fed chow, offspring fed obesogenic diet; OO, mum fed obesogenic diet, offspring fed obesogenic diet. Data are presented as means ± SEM (*n* = 7–8 from independent litters for each dietary group) and were analysed by two-way ANOVA and overall effect of post-weaning diet is reported where significant. Dunnett’s *post hoc* test for multiple comparisons test was then performed to evaluate significant differences to the CC group: ***P < 0.001.

Left ventricular (LV) ejection fraction, a measure of ventricular performance, was significantly reduced by maternal diet (two-way ANOVA, effect of maternal diet *P *=* *0.02) (*Table 2*). Similarly, LV fractional shortening was significantly reduced by maternal obesogenic diet exposure (two-way ANOVA, effect of maternal diet *P *=* *0.01) (*Table 2*). An interaction between pre- and postnatal obesogenic diet significantly affected both ejection fraction (two-way ANOVA, *P *=* *0.01) and fractional shortening (two-way ANOVA, *P *=* *0.005). This reflected the observation that an offspring obesogenic diet reduced both of these measurements in the control offspring but did not further decrease these measurements in the offspring of obese dams. Therefore, % ejection fraction and % fractional shortening were reduced by a similar magnitude in CO, OC, and OO offspring compared to the CC group. Cardiac output was similar in all four groups (*Table 2*), as was heart rate (*Table 2*). None of the other parameters measured were altered as an effect of maternal or post-weaning diet (i.e. stroke volume, LVPW, and LVID). Maternal diet-induced obesity increased offspring systolic blood pressure (two-way ANOVA, effect of maternal diet *P *=* *0.02) (*Table 2*). A postnatal obesogenic diet also increased systolic blood pressure (two-way ANOVA, *P *=* *0.05) (*Table 2*). The effects of maternal diet and offspring diet were additive, therefore, OO mice displayed the greatest increase in systolic blood pressure compared to CC (two-way ANOVA, *post hoc P *<* *0.01) (*Table 2*). Pulse rate was not different between any of the groups. Quantification of myocardial fibrosis in mid-cardiac sections revealed a significant effect of maternal diet (two-way ANOVA, *P *=* *0.0087) (*Figure 3A* and *B*), with the OO group showing the highest levels of cardiac fibrosis when compared to the other experimental groups (two-way ANOVA, *post hoc P *<* *0.01) (*Figure 3A* and *B*). In light of the development of cardiac dysfunction and fibrosis, quantification of the expression of genes implicated in the regulation of contractile and matrix remodelling was carried out, including sarco/endoplasmic reticulum Ca^2+^-ATPase (*Serca2a*) (*Figure 4A*), collagen Type I Alpha 1 (*Col1a1*) (*Figure 4B*), collagen Type III Alpha 1 (*Col3a1*) (*Figure 4C*), alpha skeletal actin (*Acta1*) (*Figure 4D*), and cardiac muscle troponin T (*Tnnt2*) (*Figure 4E*). A significant effect of offspring diet was observed on *Acta1* mRNA levels (post-weaning diet: *P *=* *0.0002), however, there was no significant effect of maternal diet (*P *=* *0.07) (*Figure 4D*). No differences as a result of maternal diet or current diet were observed in the mRNA levels of cardiac NADPH oxidases *Nox2* (*Figure 4F*) and *Nox4* (*Figure 4G*) or Nitric Oxide Synthase 3 (*Nos3*) (*Figure 4H*). There was no difference in MDA levels, a marker of lipid peroxidation, between any of the experimental groups (CC 0.34 ± 0.03, CO 0.32 ± 0.03, OC 0.34 ± 0.02, OO 0.34 ± 0.02; nmol/mg tissue).

**Table 2:** Maternal over-nutrition programmes offspring cardiac dysfunction and blood pressure independently of post-natal diet

	CC	CO	OC	OO	MD	PD	MDXPD
					*P values*		
Cardiac function							
EF (%)	73.9±3.1	55.6±1.9**	58.8±3.4*	59.3±5.2*	0.0196	0.1226	0.0136
FS (%)	42.8±2.6	28.6±1.2	30.9±2.2	31.8±3.4	0.0118	0.0942	0.0053
CO (mL/min)	18.2±1.1	15.0±1.1	16.5±1.0	17.2±1.8	0.8809	0.3433	0.1537
HR (bpm)	424.7±7.3	429.2±11.8	415.2±10.8	462.6±18.4	0.3570	0.0514	0.1037
Stroke vol. (µL)	43.2±3.2	36.7±2.6	39.6±1.8	36.7±3.2	0.5212	0.1020	0.5069
LVPW; *S* (mm)	1.3±0.04	1.2±0.04	1.1±0.07	1.3±0.1	0.3213	0.8414	0.1069
LVPW; *D* (mm)	0.8±0.04	0.8±0.03	0.8±0.04	0.9±0.06	0.7791	0.4028	0.5839
LVID; *S* (mm)	2.3±0.2	2.8±0.1	2.8±0.1	2.7±0.2	0.2291	0.2447	0.0535
LVID; *D* (mm)	3.6±0.2	3.9±0.1	3.9±0.09	3.8±0.1	0.5744	0.4917	0.2553
Blood pressure							
SBP (mm Hg)	102.2±4.7	114.6±5.0	117.6±7.7	130.8±7.5**	0.0204	0.0558	0.9482
Pulse rate (bpm)	583.7±26.9	605.1±34.3	622.5±25.5	622.3±13.8	0.6773	0.2757	0.6721

Data are presented as means ± SEM (*n* = 6–8 from independent litters for each dietary group). The effects of maternal diet (MD), postnatal diet (PD), and the interaction between the two (MDxPD) were analysed by two-way ANOVA.

CC, mum fed chow, offspring fed chow; OC, mum fed obesogenic diet, offspring fed chow; CO, mum fed chow, offspring fed obesogenic diet; OO, mum fed obesogenic diet, offspring fed obesogenic diet; EF, ejection fraction; FS, fractional shortening; CO, cardiac output; HR, heart rate; LVPW, LV posterior wall; LVID, LV internal diameter; s, measure taken in systole; d, measure taken in diastole; SBP, Systolic Blood Pressure; bpm, beat per minute.

Dunnett’s multiple comparisons test was then performed to evaluate significant differences to the CC group: *P < 0.05, **P < 0.01, ***P < 0.001.

**Figure 5:**
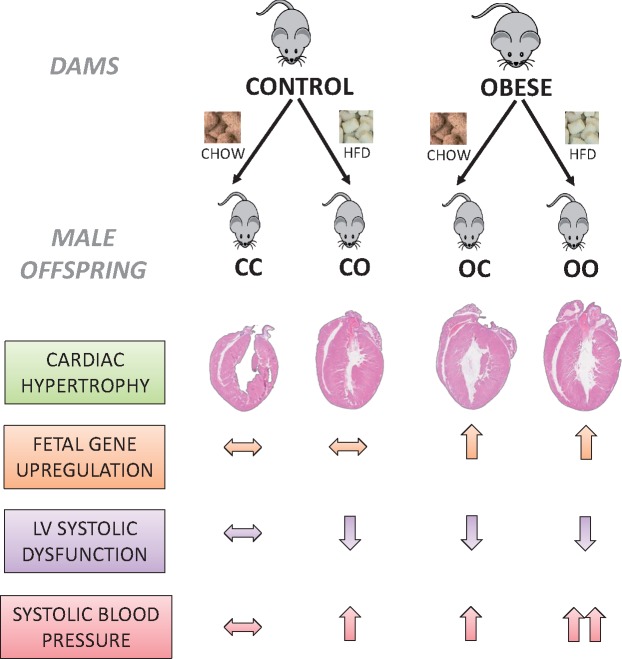
Schematic diagram of the findings of this study showing distinct programming mechanisms of hypertension and cardiac dysfunction by maternal diet-induced obesity.

## 4. Discussion

Data in this study show that: (i) Exposure to maternal obesity and current obesity caused cardiac dysfunction of comparable magnitude. The effects were not additive and therefore programming of cardiac dysfunction by maternal diet-induced obesity was independent of offspring diet; (ii) Conversely, offspring born to obese mothers developed hypertension and the effects of maternal and offspring obesity on hypertension were additive; and (iii) The offspring metabolic profile was affected by both maternal and postnatal exposure to an obesogenic diet, but at the age studied, the effects of a current obesogenic diet were greater than those of maternal obesity.

### 4.1 Effects of maternal diet on offspring cardiac structure and function

Exposure to maternal over-nutrition during gestation and lactation was sufficient to trigger pathological changes in cardiac morphology and function. Our data are in accordance with human epidemiological studies from Scottish^18^ and Finnish^19^ cohorts, which have reported that babies born to mothers with increased BMI have a higher incidence of CVD in adulthood. In the present study, mice exposed to maternal over-nutrition developed features of hypertrophic cardiomyopathy associated with increased cardiac mass and left ventricular area, and thickening of the inter-ventricular septum. A significant but small increase in ventricular cardiomyocyte area was observed. However, cardiomyocyte hypertrophy could not completely explain the increase in left ventricular area in these animals. This suggests that other potential factors, including hyperplasia of non-myocyte cells (e.g. fibroblasts) may contribute to the cardiac remodelling.

Maternal obesity programmed cardiac dysfunction in the offspring. Molecular mechanisms involved included up-regulation of *Myh7: Myh6* ratio. The increase in the *Myh7: Myh6* ratio was driven specifically by overexpression of *Myh7*, which encodes for the ventricular slow β-MHC. Transgenic over-expression of β-MHC in mouse hearts results in systolic dysfunction,^39^ likely due to a decrease in myofibrillar Ca^2+^-activated ATPase activity.^39^ Therefore, the up-regulation of *Myh7* may in part explain the reduced cardiac contractility measured in mice exposed to maternal obesity. Mice exposed to an obesogenic diet only from weaning onwards also developed contractile dysfunction but did not up-regulate their cardiac *Myh7* expression. This suggests that pathological cardiac hypertrophy is occurring only as a consequence of maternal obesity and not as a consequence of current obesity and therefore, the mechanisms underlying obesity-induced cardiac dysfunction and maternal obesity-induced cardiac dysfunction are distinct. The changes in contractile function programmed by exposure to maternal obesity were not worsened by the offspring post-weaning dietary regime. Maternal diet programmed offspring cardiac dysfunction to a similar extent as a post-weaning obesogenic diet alone, meaning that being exposed to maternal obesity during gestation is as detrimental to cardiac function in the offspring as being obese in adulthood. Consistent with the gene expression data, the lack of increased MDA levels further suggests there is no oxidative damage in the heart of any of the groups at the age studied. Despite the significant reduction in cardiac systolic function, indexed by impaired ejection fraction and fractional shortening in offspring of obese dams, cardiac output was maintained. The reported increase in cardiac sympathetic dominance in hearts of offspring of obese mothers may help maintain cardiac output and appropriate systemic perfusion despite impaired cardiac systolic function in these mice.^14^ Our data are in contrast to a previous study where the authors investigated the consequences of maternal and postnatal high-fat diet on offspring cardiac function.^40^ Turdi and colleagues reported a loss of contractile function upon maternal and weaning high-fat diet exposure, whereas exposure to maternal high-fat diet alone did not programme any significant changes in cardiac performance.^40^ We found that both offspring groups exposed to a maternal obesogenic diet develop a significant reduction in cardiac function, regardless of their post-weaning diet. Such phenotypic differences in our and their study could be attributed to the composition of the experimental diets (high-fat–high-sugar diet vs. high-fat diet only in the Turdi study). The high-fat diet (45% energy from fat) used by Turdi and colleagues induced only modest changes in maternal body weight whereas in our model, dams fed the palatable obesogenic diet were measurably obese at mating and insulin resistant throughout pregnancy compared to lean dams.^12^^,^^41^ It is likely that a lower degree of obesity and modest metabolic alterations in the mothers is not sufficient to induce programming effects in chow-fed offspring at a young age. It is becoming apparent that not only the degree of obesity and the consumption of fat *per se* but also the *in utero* metabolic *milieu* (in particular insulin resistance) is important in the programming of cardiometabolic disturbances in humans^20^ and animal models.^41^^,^^42^

### 4.2 Effects of maternal diet on offspring arterial blood pressure

Maternal obesity programmed hypertension in the adult offspring. Hypertension is a known programmed phenotype in animals exposed to maternal diet-induced obesity^12^^,^^43^ and is known to be increased as a consequence of current obesity.^44^ The increase in blood pressure as a consequence of consuming an obesogenic diet postnatal diet is likely driven by a combination of hyperinsulinaemia and hyperleptinaemia which cause an increase in peripheral vascular resistance and therefore blood pressure. We found the greatest increase in systolic blood pressure in mice exposed to a combined maternal and post-weaning obesogenic diet, consistent with other studies in non-human primates^9^ and rodents.^28^^,^^40^ However, these offspring showed no change in cardiac output. This dissociation is consistent with peripheral vascular resistance contributing to the hypertension in these offspring. Several studies of adverse intrauterine conditions showing programmed hypertension in the offspring, including maternal obesity, also reported a peripheral vasoconstrictor phenotype in the offspring, including reduced endothelium-dependent relaxation^36^^,^^45^ with impaired nitric oxide bioavailability,^7^^,^^28^^,^^36^ dysregulation of the renin–angiotensin system,^46^ and sympathetic over-activation driven by maternal hyperleptinaemia.^47^ In the present study, it is plausible that chronic hyperinsulinaemia and/or hyperleptinaemia in the ‘double-exposed’ group would further promote vascular endothelial dysfunction, leading to a worsening of peripheral vascular resistance and thus further increases in blood pressure. An increase in arterial blood pressure but not in heart rate in the adult offspring suggests that the arterial baroreflex function is reset to allow an elevated resting arterial blood pressure without a concomitant fall in heart rate. In adult individuals, persistent resetting of arterial baroreflex function is a hallmark of essential hypertension.^48^ Resetting of arterial baroreflex function has been reported in the fetus in an ovine model of pregnancy exposed to synthetic glucocorticoids in late gestation.^49^ Therefore, data in the present manuscript suggest that other forms of adversity during critical windows of development, such as maternal diet-induced obesity during pregnancy as well as a postnatal obesogenic diet can not only programme hypertension in later life but also a shift in the set point of the arterial baroreflex to operate at an elevated basal arterial blood pressure. Further, these effects of maternal and offspring exposure to an obesogenic diet occur synergistically, perpetuating susceptibility to progressive hypertension in the adult offspring. An increase in peripheral vascular resistance will increase cardiac afterload, which can promote left ventricular remodelling.^50^ A significant up-regulation of cardiac *Acta1* mRNA levels was observed in mice weaned onto an obesogenic diet, which may underlie a compensatory response to offset the increase in cardiac afterload, also helping to maintain cardiac output.

### 4.3 Interaction between maternal and post-weaning diet on offspring metabolism

Early exposure to pre-gestational and gestational obesity is known to programme metabolic disturbances in the next generation in humans^51^ and animals.^52^ In the current study mice exposed to maternal over-nutrition during gestation and lactation but fed a chow diet post-weaning develop modest metabolic perturbations at this age. It is likely that such disturbances, in particular insulin and leptin levels, will be exacerbated with ageing. They also showed increased adiposity but overall no difference in body weight,^14^^,^^29^ suggesting that these mice may be prone to develop obesity later on. When these animals were weaned onto an obesogenic diet they displayed a significant increase in body weight as well as augmented body fat accretion, which resulted in the greatest increase in circulating levels of insulin and leptin. The effects of maternal obesity and an offspring obesogenic diet on hyperinsulinaemia and hyperleptinaemia were additive. Adiponectin levels were not affected by either maternal obesity or a post-weaning obesogenic diet, however, resistin levels were raised but only as an effect of post-weaning diet. This is consistent with previous reports.^53^^,^^54^ Therefore, programmed changes in blood pressure and cardiac function are not mediated by changes in adiponectin or resistin. However, an interplay between insulin and resistin may contribute to the increase in blood pressure in the OO group.^55^ Thus, the double exposure to an obesogenic diet during these two periods of life would increase their vulnerability to metabolic dysfunction, as previously reported.^24^^,^^25^ This is consistent with the observation that not everyone living in the same obesogenic environment is equally vulnerable to its detrimental effects. Offspring exposed to maternal obesity represent one vulnerable group as they already display modest metabolic dysfunction that is then increased by a second ‘hit’.

### 4.4 Windows of developmental programming

Our findings suggest that the programming of hypertension and cardiac dysfunction are, at least initially, programmed via different mechanisms. One possibility is that they occur during different critical time periods. In the current study, exposure to maternal obesity occurred during pregnancy and lactation. It is not clear when precisely during this time period of development the programming of cardiac dysfunction and hypertension occurs.

We have shown that 8-week-old mice exposed to maternal obesity developed left ventricular cardiac hypertrophy associated with hyperinsulinaemia.^29^ In addition, we have recently reported that maternal hyperinsulinaemia is a predictor of insulin resistance in the offspring, and a mild regime of exercise confined to the gestation period (mating to embryonic day 17) prevented maternal hyperinsulinaemia and offspring hyperinsulinaemia.^41^ Since exercise in obese mothers modifies the risk of offspring developing hyperinsulinaemia, it can be hypothesized that the cardiovascular risk may be programmed *in utero* and is therefore modifiable by interventions during pregnancy. This is supported by previous studies in an ovine model of maternal obesity showing that alterations in cardiac insulin signalling and impaired response to cardiac work load stress are already present during fetal life.^56^ Furthermore it has been shown that injection of insulin at day 19 of gestation in the rat leads to increased cardiac protein synthesis (a hallmark of cardiac hypertrophy).^57^

The lactational period has been shown to be a critical time window for programming of hypertension.^47^^,^^58^ Using the same mouse model as in this study, Samuelsson et al. have showed that maternal obesity results in maternal hyperleptinaemia and programmes hypertension of sympathetic origin in the offspring due to leptin-induced over-activation of the central melanocortin system in lactating mice.^47^ Indeed, mice with a specific deletion of melanocortin-4 receptors in the paraventricular nucleus of the hypothalamus were protected from sympathetic over-activation by maternal leptin and consequently hypertension.^47^ These observations identified the central melanocortin system as a key mediator of the programming of hypertension by maternal diet-induced obesity and are consistent with our data whereby maternal obesity programmes a hypertensive phenotype that is exacerbated when the mice are maintained on the obesogenic diet after weaning.

### 4.5 Limitations

In this study, we measured only the gene expression of *Nox2*, *Nox4*, and *Nos3* at the mRNA level which were not different between groups. However, mRNA levels do not necessarily reflect activity levels of these enzymes and therefore we can make no conclusions regarding the activity of the NOX enzymes or NOS coupling or activity. Although there were no differences between groups in MDA (a marker of lipid peroxidation), this obviously reflects a balance between ROS generation, anti-oxidant defences, and tissue repair/turnover. Therefore, the possibility still remains that there are differences in individual enzymatic sources of ROS.

In conclusion, exposure to maternal diet-induced obesity was sufficient to programme hypertension, left ventricular pathological remodelling, and ventricular dysfunction in the offspring. Molecular mechanisms involved in mediating changes in cardiac structure and function included the transcriptional re-activation of cardiac fetal genes as well as genes involved in the regulation of contractile function and matrix remodelling in the adult heart (*Figure 5*). A post-weaning obesogenic diet coupled with a maternal exposure to the same diet worsened offspring hyperinsulininaemia, hyperleptinaemia, fat accretion, hypertension risk, and cardiac fibrosis. In contrast, the post-weaning obesogenic diet caused cardiac dysfunction in control offspring but did not worsen the programmed cardiac dysfunction in the offspring of obese dams. Thus, obesity *per se* can act synergistically with maternal obesity to further increase circulating insulin and leptin levels, which may contribute to hypertension risk but not to additional loss of cardiac function. The findings suggest that the effect of obesity during pregnancy is of a similar magnitude to that of current obesity on cardiac dysfunction and thus highlight the importance of this period as a target for interventions aimed at reducing CVD. 

## Supplementary material

Supplementary material is available at *Cardiovascular Research* online.

## Supplementary Material

Supplementary Data

## References

[1] Lauby-Secretan B, Scoccianti C, Loomis D, Grosse Y, Bianchini F, Straif K, Group IAfRoCHW. Body fatness and cancer–viewpoint of the IARC Working Group. N Engl J Med 2016;375:794–798. 27557308 10.1056/NEJMsr1606602 PMC6754861

[2] Townsend N, Wilson L, Bhatnagar P, Wickramasinghe K, Rayner M, Nichols M. Cardiovascular disease in Europe: epidemiological update 2016. Eur H J 2016;37:3232–3245. 10.1093/eurheartj/ehw334 PMC13480964 27523477

[3] Poston L, Caleyachetty R, Cnattingius S, Corvalán C, Uauy R, Herring S, Gillman MW. Preconceptional and maternal obesity: epidemiology and health consequences. Lancet Diabetes Endocrinol 2016;4:1025–1036. 27743975 10.1016/S2213-8587(16)30217-0

[4] Zambrano E, Ibáñez C, Martínez-Samayoa PM, Lomas-Soria C, Durand-Carbajal M, Rodríguez-González GL. Maternal obesity: lifelong metabolic outcomes for offspring from poor developmental trajectories during the perinatal period. Arch Med Res 2016;47:1–12. 26827819 10.1016/j.arcmed.2016.01.004

[5] Gluckman PD, Hanson MA, Cooper C, Thornburg KL. Effect of in utero and early-life conditions on adult health and disease. N Engl J Med 2008;359:61–73. 18596274 10.1056/NEJMra0708473 PMC3923653

[6] Martin-Gronert MS, Ozanne SE. Mechanisms underlying the developmental origins of disease. Rev Endocr Metab Disord 2012;13:85–92. 22430227 10.1007/s11154-012-9210-z

[7] Giussani DA, Davidge ST. Developmental programming of cardiovascular disease by prenatal hypoxia. J Dev Orig Health Dis 2013;4:328–337. 24970726 10.1017/S204017441300010X

[8] Godfrey KM, Reynolds RM, Prescott SL, Nyirenda M, Jaddoe VW, Eriksson JG, Broekman BF. Influence of maternal obesity on the long-term health of offspring. Lancet Diabetes Endocrinol 2017;5:53–64. 27743978 10.1016/S2213-8587(16)30107-3 PMC5245733

[9] Fan L, Lindsley SR, Comstock SM, Takahashi DL, Evans AE, He GW, Thornburg KL, Grove KL. Maternal high-fat diet impacts endothelial function in nonhuman primate offspring. Int J Obes 2013;37:254–262. 10.1038/ijo.2012.42 PMC3468685 22450853

[10] Fan X, Turdi S, Ford SP, Hua Y, Nijland MJ, Zhu M, Nathanielsz PW, Ren J. Influence of gestational overfeeding on cardiac morphometry and hypertrophic protein markers in fetal sheep. J Nutr Biochem 2011;22:30–37. 20188535 10.1016/j.jnutbio.2009.11.006 PMC2901772

[11] Liang C, Oest ME, Prater MR. Intrauterine exposure to high saturated fat diet elevates risk of adult-onset chronic diseases in C57BL/6 mice. Birth Defect Res B 2009;86:377–384. 10.1002/bdrb.20206 19750488

[12] Samuelsson AM, Matthews PA, Argenton M, Christie MR, McConnell JM, Jansen EH, Piersma AH, Ozanne SE, Twinn DF, Remacle C, Rowlerson A, Poston L, Taylor PD. Diet-induced obesity in female mice leads to offspring hyperphagia, adiposity, hypertension, and insulin resistance: a novel murine model of developmental programming. Hypertension 2008;51:383–392. 18086952 10.1161/HYPERTENSIONAHA.107.101477

[13] Huang Y, Yan X, Zhao JX, Zhu MJ, McCormick RJ, Ford SP, Nathanielsz PW, Ren J, Du M. Maternal obesity induces fibrosis in fetal myocardium of sheep. Am J Physiol Endocrinol Metab 2010;299:E968–E975. 20876759 10.1152/ajpendo.00434.2010 PMC3006252

[14] Blackmore HL, Niu Y, Fernandez-Twinn DS, Tarry-Adkins JL, Giussani DA, Ozanne SE. Maternal diet-induced obesity programs cardiovascular dysfunction in adult male mouse offspring independent of current body weight. Endocrinology 2014;155:3970–3980. 25051449 10.1210/en.2014-1383 PMC4255219

[15] Taylor PD, McConnell J, Khan IY, Holemans K, Lawrence KM, Asare-Anane H, Persaud SJ, Jones PM, Petrie L, Hanson MA, Poston L. Impaired glucose homeostasis and mitochondrial abnormalities in offspring of rats fed a fat-rich diet in pregnancy. Am J Physiol Regul Integr Comp Physiol 2005;288:R134–R139. 15388492 10.1152/ajpregu.00355.2004

[16] Volpato AM, Schultz A, Magalhães-da-Costa E, Correia ML, Águila MB, Mandarim-de-Lacerda CA. Maternal high-fat diet programs for metabolic disturbances in offspring despite leptin sensitivity. Neuroendocrinology 2012;96:272–284. 22456428 10.1159/000336377

[17] Janoschek R, Bae-Gartz I, Vohlen C, Alcázar MA, Dinger K, Appel S, Dötsch J, Hucklenbruch-Rother E. Dietary intervention in obese dams protects male offspring from WAT induction of TRPV4, adiposity, and hyperinsulinemia. Obesity (Silver Spring) 2016;24:1266–1273. 27106804 10.1002/oby.21486

[18] Reynolds RM, Allan KM, Raja EA, Bhattacharya S, McNeill G, Hannaford PC, Sarwar N, Lee AJ, Bhattacharya S, Norman JE. Maternal obesity during pregnancy and premature mortality from cardiovascular event in adult offspring: follow-up of 1 323 275 person years. BMJ 2013;347:f4539. 23943697 10.1136/bmj.f4539 PMC3805484

[19] Eriksson JG, Sandboge S, Salonen MK, Kajantie E, Osmond C. Long-term consequences of maternal overweight in pregnancy on offspring later health: findings from the Helsinki Birth Cohort Study. Ann Med 2014;46:434–438. 24910160 10.3109/07853890.2014.919728

[20] Guénard F, Deshaies Y, Cianflone K, Kral JG, Marceau P, Vohl MC. Differential methylation in glucoregulatory genes of offspring born before vs. after maternal gastrointestinal bypass surgery. Proc Natl Acad Sci U S A 2013;110:11439–11444. 23716672 10.1073/pnas.1216959110 PMC3710842

[21] Ingul CB, Lorås L, Tegnander E, Eik-Nes SH, Brantberg A. Maternal obesity affects foetal myocardial function already in first trimester. Ultrasound Obstet Gynecol 2016;47:433–442. 25761057 10.1002/uog.14841

[22] Catalano PM, Presley L, Minium J, Hauguel-de Mouzon S. Fetuses of obese mothers develop insulin resistance in utero. Diabetes Care 2009;32:1076–1080. 19460915 10.2337/dc08-2077 PMC2681036

[23] Schrempft S, van Jaarsveld CH, Fisher A, Fildes A, Wardle J. Maternal characteristics associated with the obesogenic quality of the home environment in early childhood. Appetite 2016;107:392–397. 27554185 10.1016/j.appet.2016.08.108

[24] Howie GJ, Sloboda DM, Kamal T, Vickers MH. Maternal nutritional history predicts obesity in adult offspring independent of postnatal diet. J Physiol 2009;587:905–915. 19103681 10.1113/jphysiol.2008.163477 PMC2669979

[25] Chen H, Simar D, Ting JH, Erkelens JR, Morris MJ. Leucine improves glucose and lipid status in offspring from obese dams, dependent on diet type, but not caloric intake. J Neuroendocrinol 2012;24:1356–1364. 22612562 10.1111/j.1365-2826.2012.02339.x

[26] Benkalfat NB, Merzouk H, Bouanane S, Merzouk SA, Bellenger J, Gresti J, Tessier C, Narce M. Altered adipose tissue metabolism in offspring of dietary obese rat dams. Clin Sci 2011;121:19–28. 21288203 10.1042/CS20100534

[27] de Almeida Faria J, Duque-Guimarães D, Carpenter AA, Loche E, Ozanne SE. A post-weaning obesogenic diet exacerbates the detrimental effects of maternal obesity on offspring insulin signaling in adipose tissue. Sci Rep 2017;7:44949. 28338072 10.1038/srep44949 PMC5364470

[28] Torrens C, Ethirajan P, Bruce KD, Cagampang FR, Siow RC, Hanson MA, Byrne CD, Mann GE, Clough GF. Interaction between maternal and offspring diet to impair vascular function and oxidative balance in high fat fed male mice. PLoS One 2012;7:e50671. 23227196 10.1371/journal.pone.0050671 PMC3515587

[29] Fernandez-Twinn DS, Blackmore HL, Siggens L, Giussani DA, Cross CM, Foo R, Ozanne SE. The programming of cardiac hypertrophy in the offspring by maternal obesity is associated with hyperinsulinemia, AKT, ERK, and mTOR activation. Endocrinology 2012;153:5961–5971. 23070543 10.1210/en.2012-1508 PMC3568261

[30] Shimizu I, Minamino T. Physiological and pathological cardiac hypertrophy. J Mol Cell Cardiol 2016;97:245–262. 27262674 10.1016/j.yjmcc.2016.06.001

[31] Heineke J, Molkentin JD. Regulation of cardiac hypertrophy by intracellular signalling pathways. Nat Rev Mol Cell Biol 2006;7:589–600. 16936699 10.1038/nrm1983

[32] Dirkx E, da Costa Martins PA, De Windt LJ. Regulation of fetal gene expression in heart failure. Biochim Biophys Acta 2013;1832:2414–2424. 24036209 10.1016/j.bbadis.2013.07.023

[33] Periasamy M, Bhupathy P, Babu GJ. Regulation of sarcoplasmic reticulum Ca2+ ATPase pump expression and its relevance to cardiac muscle physiology and pathology. Cardiovasc Res 2008;77:265–273. 18006443 10.1093/cvr/cvm056

[34] Lombardi R, Betocchi S, Losi MA, Tocchetti CG, Aversa M, Miranda M, D’Alessandro G, Cacace A, Ciampi Q, Chiariello M. Myocardial collagen turnover in hypertrophic cardiomyopathy. Circulation 2003;108:1455–1460. 12952838 10.1161/01.CIR.0000090687.97972.10

[35] Schmittgen TD, Livak KJ. Analyzing real-time PCR data by the comparative C(T) method. Nat Protoc 2008;3:1101–1108. 18546601 10.1038/nprot.2008.73

[36] Giussani DA, Camm EJ, Niu Y, Richter HG, Blanco CE, Gottschalk R, Blake EZ, Horder KA, Thakor AS, Hansell JA, Kane AD, Wooding FB, Cross CM, Herrera EA. Developmental programming of cardiovascular dysfunction by prenatal hypoxia and oxidative stress. PLoS One 2012;7:e31017. 22348036 10.1371/journal.pone.0031017 PMC3278440

[37] Liang X, Yang Q, Zhang L, Maricelli JW, Rodgers BD, Zhu MJ, Du M. Maternal high-fat diet during lactation impairs thermogenic function of brown adipose tissue in offspring mice. Sci Rep 2016;6:34345. 27686741 10.1038/srep34345 PMC5043374

[38] Whittle AJ, Carobbio S, Martins L, Slawik M, Hondares E, Vázquez MJ, Morgan D, Csikasz RI, Gallego R, Rodriguez-Cuenca S, Dale M, Virtue S, Villarroya F, Cannon B, Rahmouni K, López M, Vidal-Puig A. BMP8B increases brown adipose tissue thermogenesis through both central and peripheral actions. Cell 2012;149:871–885. 22579288 10.1016/j.cell.2012.02.066 PMC3383997

[39] Tardiff JC, Hewett TE, Factor SM, Vikstrom KL, Robbins J, Leinwand LA. Expression of the beta (slow)-isoform of MHC in the adult mouse heart causes dominant-negative functional effects. Am J Physiol Heart Circ Physiol 2000;278:H412–H419. 10666070 10.1152/ajpheart.2000.278.2.H412

[40] Turdi S, Ge W, Hu N, Bradley KM, Wang X, Ren J. Interaction between maternal and postnatal high fat diet leads to a greater risk of myocardial dysfunction in offspring via enhanced lipotoxicity, IRS-1 serine phosphorylation and mitochondrial defects. J Mol Cell Cardiol 2013;55:117–129. 23266593 10.1016/j.yjmcc.2012.12.007

[41] Fernandez-Twinn DS, Gascoin G, Musial B, Carr S, Duque-Guimaraes D, Blackmore HL, Alfaradhi MZ, Loche E, Sferruzzi-Perri AN, Fowden AL, Ozanne SE. Exercise rescues obese mothers’ insulin sensitivity, placental hypoxia and male offspring insulin sensitivity. Sci Rep 2017;7:44650. 28291256 10.1038/srep44650 PMC5349590

[42] Isganaitis E, Woo M, Ma H, Chen M, Kong W, Lytras A, Sales V, Decoste-Lopez J, Lee KJ, Leatherwood C, Lee D, Fitzpatrick C, Gall W, Watkins S, Patti ME. Developmental programming by maternal insulin resistance: hyperinsulinemia, glucose intolerance, and dysregulated lipid metabolism in male offspring of insulin-resistant mice. Diabetes 2014;63:688–700. 24186867 10.2337/db13-0558 PMC3900545

[43] Elahi MM, Cagampang FR, Mukhtar D, Anthony FW, Ohri SK, Hanson MA. Long-term maternal high-fat feeding from weaning through pregnancy and lactation predisposes offspring to hypertension, raised plasma lipids and fatty liver in mice. Br J Nutr 2009;102:514–519. 19203419 10.1017/S000711450820749X

[44] Rahmouni K, Correia ML, Haynes WG, Mark AL. Obesity-associated hypertension: new insights into mechanisms. Hypertension 2005;45:9–14. 15583075 10.1161/01.HYP.0000151325.83008.b4

[45] Armitage JA, Lakasing L, Taylor PD, Balachandran AA, Jensen RI, Dekou V, Ashton N, Nyengaard JR, Poston L. Developmental programming of aortic and renal structure in offspring of rats fed fat-rich diets in pregnancy. J Physiol (Lond) 2005;565:171–184. 15774514 10.1113/jphysiol.2005.084947 PMC1464506

[46] Guberman C, Jellyman JK, Han G, Ross MG, Desai M. Maternal high-fat diet programs rat offspring hypertension and activates the adipose renin-angiotensin system. Am J Obstet Gynecol 2013;209:262.e1–268. 23743273 10.1016/j.ajog.2013.05.023 PMC4010310

[47] Samuelsson AS, Mullier A, Maicas N, Oosterhuis NR, Eun Bae S, Novoselova TV, Chan LF, Pombo JM, Taylor PD, Joles JA, Coen CW, Balthasar N, Poston L. Central role for melanocortin-4 receptors in offspring hypertension arising from maternal obesity. Proc Natl Acad Sci U S A 2016;113:12298–12303. 27791019 10.1073/pnas.1607464113 PMC5087049

[48] Xie PL, McDowell TS, Chapleau MW, Hajduczok G, Abboud FM. Rapid baroreceptor resetting in chronic hypertension. Implications for normalization of arterial pressure. Hypertension 1991;17:72–79. 1986984 10.1161/01.hyp.17.1.72

[49] Fletcher AJ, McGarrigle HH, Edwards CM, Fowden AL, Giussani DA. Effects of low dose dexamethasone treatment on basal cardiovascular and endocrine function in fetal sheep during late gestation. J Physiol (Lond) 2002;545:649–660. 12456840 10.1113/jphysiol.2001.015693 PMC2290705

[50] Kahan T, Bergfeldt L. Left ventricular hypertrophy in hypertension: its arrhythmogenic potential. Heart 2005;91:250–256. 15657259 10.1136/hrt.2004.042473 PMC1768675

[51] Catalano P, deMouzon SH. Maternal obesity and metabolic risk to the offspring: why lifestyle interventions may have not achieved the desired outcomes. Int J Obes 2015;39:642–649. 10.1038/ijo.2015.15 PMC4700513 25777180

[52] Williams L, Seki Y, Vuguin PM, Charron MJ. Animal models of in utero exposure to a high fat diet: a review. Biochim Biophys Acta 2014;1842:507–519. 23872578 10.1016/j.bbadis.2013.07.006 PMC3895417

[53] Muse ED, Obici S, Bhanot S, Monia BP, McKay RA, Rajala MW, Scherer PE, Rossetti L. Role of resistin in diet-induced hepatic insulin resistance. J Clin Invest 2004;114:232–239. 15254590 10.1172/JCI21270 PMC449748

[54] Rajala MW, Qi Y, Patel HR, Takahashi N, Banerjee R, Pajvani UB, Sinha MK, Gingerich RL, Scherer PE, Ahima RS. Regulation of resistin expression and circulating levels in obesity, diabetes, and fasting. Diabetes 2004;53:1671–1679. 15220189 10.2337/diabetes.53.7.1671

[55] Gentile MT, Vecchione C, Marino G, Aretini A, Di Pardo A, Antenucci G, Maffei A, Cifelli G, Iorio L, Landolfi A, Frati G, Lembo G. Resistin impairs insulin-evoked vasodilation. Diabetes 2008;57:577–583. 18065520 10.2337/db07-0557

[56] Wang J, Ma H, Tong C, Zhang H, Lawlis GB, Li Y, Zang M, Ren J, Nijland MJ, Ford SP, Nathanielsz PW, Li J. Overnutrition and maternal obesity in sheep pregnancy alter the JNK-IRS-1 signaling cascades and cardiac function in the fetal heart. Faseb J 2010;24:2066–2076. 20110268 10.1096/fj.09-142315 PMC2874473

[57] Johnson JD, Dunham T, Wogenrich FJ, Greenberg RE, Loftfield RB, Skipper BJ. Fetal hyperinsulinemia and protein turnover in fetal rat tissues. Diabetes 1990;39:541–548. 1691995 10.2337/diab.39.5.541

[58] Vieira AK, Soares VM, Bernardo AF, Neves FA, Mattos AB, Guedes RM, Cortez E, Andrade DC, Lacerda-Miranda G, Garcia-Souza EP, Moura AS. Overnourishment during lactation induces metabolic and haemodynamic heart impairment during adulthood. Nutr Metab Cardiovasc Dis 2015;25:1062–1069. 26315623 10.1016/j.numecd.2015.07.009

