# Supplementary material for: Maternal diet-induced obesity programmes cardiac dysfunction in male mice independently of post-weaning diet
Source: Cardiovasc Res. 2018 Apr 4;114(10):1372–84. doi: 10.1093/cvr/cvy082 (PMC6054211; doi:10.1093/cvr/cvy082)

Supplementary Table 1. Primer sequences

Gene name	Forward (5'->3')	Reverse (3'->5')
Nppa	GGAGCCTACGAAGATCCAGC	TTCGGTACCGGAAGCTGTTG
Nppb	GGTCCAGCAGAGACCTCAAA	CAACTTCAGTGCGTTACAGCC
Myh6	CTGTTCCTCTCTCCGTCCAG	ATTCTGTCACTCAAACTCTGGTTA
Myh7	CGCATCAAGGAGCTCACC	CTGCAGCCGCAGTAGGTT
Acta1	CAGAGTCAGAGCAGCAGAAACT	AGCCGTTGTCACACACAAGA
Serca2a	TTTGCCGCTCATTTTCCAGA	GGCTGCACACACTCTTTACC
Tnnt2	AGCCCACATGCCTGCTTAAA	TCTCGGCTCTCCCTCTGAAC
Col1a1	CGATGGATTCCCGTTCGAGT	CGATCTCGTTGGATCCCTGG
Col3a1	GAGGGCCATAGCTGAACTGA	TGACTGTCCCACGTAAGCAC
Nox2	GGGAACTGGGCTGTGAATGA	CAGTGCTGACCCAAGGAGTT
Nox4	CCAAATGTTGGGCGATTGTGT	CAGGACTGTCCGGCACATAG
Nos3	Taqman Probe	mm00435204_m1
Ucp1	CCCGCTGGACACTGCC	ACCTAATGGTACTGGAAGCCTGG
Ppargc1a	TCTTTGCAGTGTGCTGCTCT	CGCTACACCACTTCAATCCA
Ppara	TGCAGCCTCAGCCAAGTTGAA	CCCGAACTTGACCAGCCACA
Pparg	TTTAAAAACAAGACTACCCTTTACTGAAATT	AGAGGTCCACAGAGCTGATTCC
Gapdh	CAACGGGAAGCCCATCAC	GCCTCACCCCATTTGATGTT
Sdha	TTCCGTGTGGGGAGTGTATTG	ATTCTGCAGCTCCAGGGTCTC
36b4	AGATGCAGCAGATCCGCAT	GTTCTTGCCCATCAGCACC

Supplementary Table 2.

	CC	CO	OC	OO	MD	PD	MDXPD
					***P*-values**		
Mesenteric (g)	0.17±0.01	0.30±0.06	0.21±0.02	0.33±0.05*	0.4207	0.0047	0.8767
as % body weight	0.70±0.05	1.02±0.2	0.85±0.1	1.06±0.1	0.4111	0.0360	0.6629
Epididymal (g)	0.24±0.03	0.94±0.15**	0.40±0.025	1.15±0.23***	0.1674	<0.0001	0.8680
as % body weight	0.95±0.1	3.2±0.4***	1.62± 0.1	3.7±0.5***	0.1087	<0.0001	0.8849
Retroperitoneal (g)	0.05±0.008	0.27±0.05***	0.10±0.02	0.26±0.05**	0.6661	<0.0001	0.3590
as % body weight	0.20±0.03	0.92±0.2***	0.41±0.07	0.80±0.1**	0.6924	<0.0001	0.1447
BAT	0.07±0.006	0.12±0.01**	0.08±0.004	0.17±0.02***	0.0280	<0.0001	0.1039
as % body weight	0.29±0.02	0.42±0.03*	0.32±0.02	0.55±0.05***	0.0159	<0.0001	0.1098

Supplementary Figure 1.

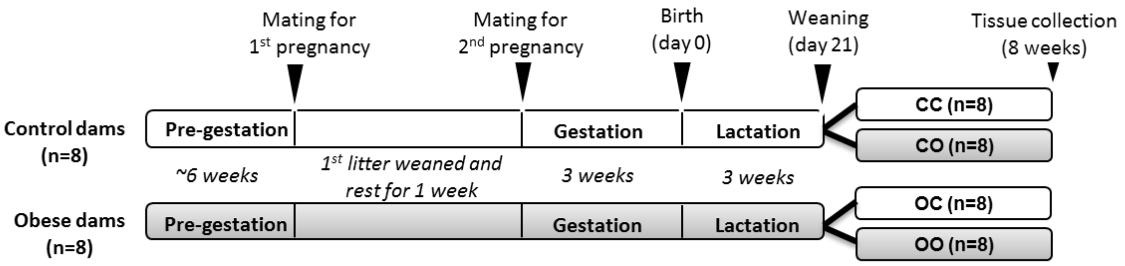

Supplementary Figure 2.

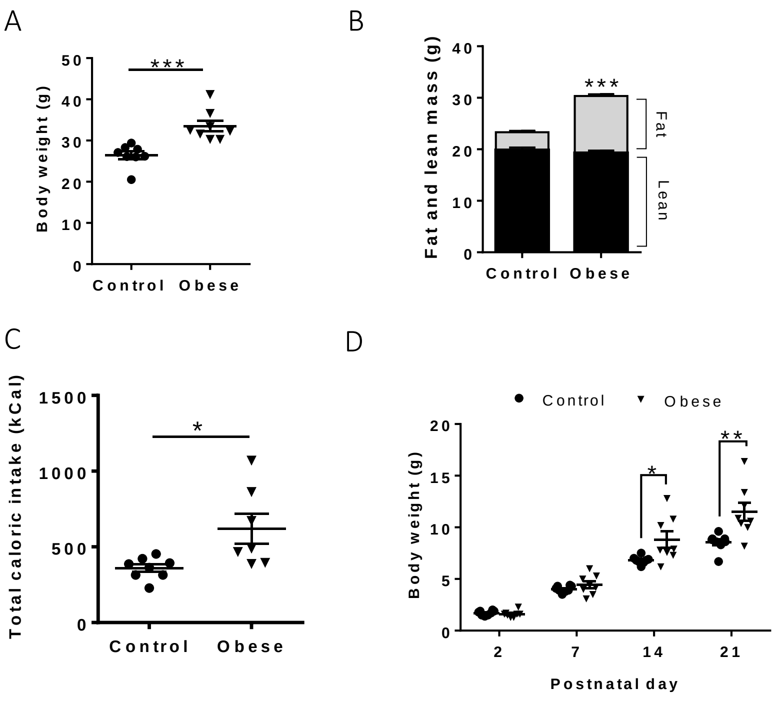

Supplementary Figure 3.

Brown Adipose

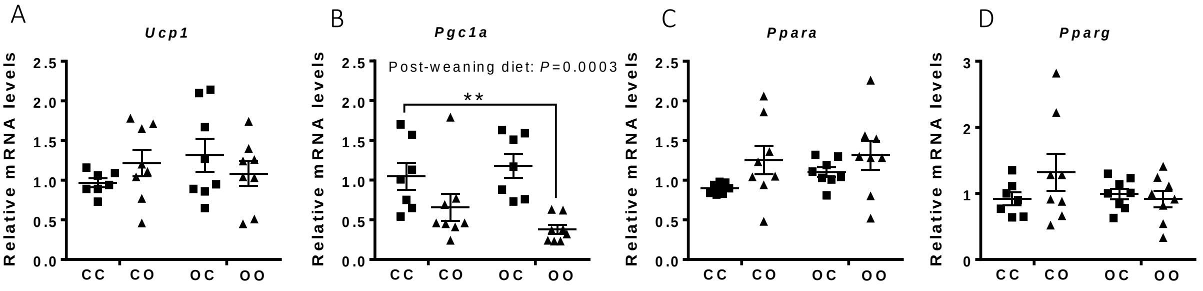

Subcutaneous Adipose

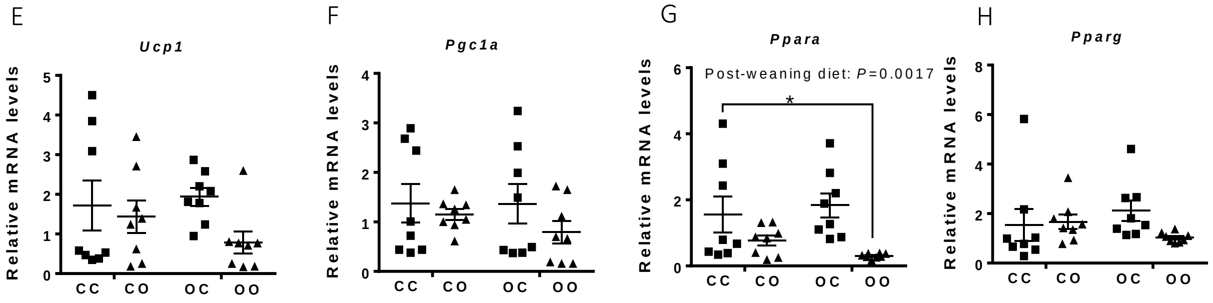

Supplement: Supplementary Data [file cvy082_supp.pdf]
